# Age‐Trajectories of Higher‐Order Diffusion Properties of Major Brain Metabolites in Cerebral and Cerebellar Gray Matter Using In Vivo Diffusion‐Weighted MR Spectroscopy at 3T

**DOI:** 10.1111/acel.14477

**Published:** 2025-01-16

**Authors:** Kadir Şimşek, Cécile Gallea, Guglielmo Genovese, Stephane Lehéricy, Francesca Branzoli, Marco Palombo

**Affiliations:** ^1^ Cardiff University Brain Research Imaging Centre (CUBRIC), School of Psychology Cardiff University Cardiff UK; ^2^ School of Computer Science and Informatics Cardiff University Cardiff UK; ^3^ Paris Brain Institute – ICM Team “Movement Investigations and Therapeutics” Paris France; ^4^ Paris Brain Institute – ICM, INSERM U 1127, CNRS UMR 7225 Sorbonne University Paris France; ^5^ Department of Neuromedicine and Movement Science Norwegian University of Science and Technology Trondheim Norway

**Keywords:** aging, cerebellum, cerebral cortex, diffusion modeling, gray matter, magnetic resonance spectroscopy, metabolite

## Abstract

Healthy brain aging involves changes in both brain structure and function, including alterations in cellular composition and microstructure across brain regions. Unlike diffusion‐weighted MRI (dMRI), diffusion‐weighted MR spectroscopy (dMRS) can assess cell‐type specific microstructural changes, providing indirect information on both cell composition and microstructure through the quantification and interpretation of metabolites' diffusion properties. This work investigates age‐related changes in the higher‐order diffusion properties of total N‐Acetyl‐aspartate (neuronal biomarker), total choline (glial biomarker), and total creatine (both neuronal and glial biomarker) beyond the classical apparent diffusion coefficient in cerebral and cerebellar gray matter of healthy human brain. Twenty‐five subjects were recruited and scanned using a diffusion‐weighted semi‐LASER sequence in two brain regions‐of‐interest (ROI) at 3T: posterior‐cingulate (PCC) and cerebellar cortices. Metabolites' diffusion was characterized by quantifying metrics from both Gaussian and non‐Gaussian signal representations and biophysical models. All studied metabolites exhibited lower apparent diffusivities and higher apparent kurtosis values in the cerebellum compared to the PCC, likely stemming from the higher microstructural complexity of cellular composition in the cerebellum. Multivariate regression analysis (accounting for ROI tissue composition as a covariate) showed slight decrease (or no change) of all metabolites' diffusivities and slight increase of all metabolites' kurtosis with age, none of which statistically significant (*p* > 0.05). The proposed age‐trajectories provide benchmarks for identifying anomalies in the diffusion properties of major brain metabolites which could be related to pathological mechanisms altering both the brain microstructure and cellular composition.

## Graphical abstract

This study examines age‐related changes in the diffusion properties of metabolites in cerebral and cerebellar gray matter. The cerebellum showed lower diffusivity and higher kurtosis, suggesting higher microstructural complexity. Multivariate regression revealed slight decreases in diffusivity and slight increases in kurtosis with age, but these changes were not significant. Age‐trajectories of diffusion properties may serve as benchmark to detect pathological brain microstructural changes.

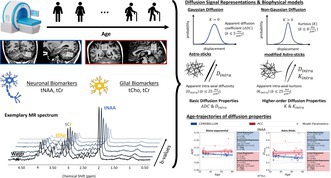

## 1 Introduction

Healthy aging involves numerous and heterogeneous functional and structural changes in the brain depending also on the considered anatomical region. For instance, in vivo studies showed that the cerebellum presents slower age‐related morphological changes compared to the cerebral cortex (Liang and Carlson 2020), possibly due to different microstructural properties. Indeed, the cerebellum contains 60% to 80% of the total amount of neurons in the brain for only 10% of the brain mass (Colin, Ris, and Godaux 2001; Walløe, Pakkenberg, and Fabricius 2014). Investigating the neurobiological underpinnings of aging in the cerebellum is of interest as this structure projects to the entire brain and mediates cognitive functions affected by aging (Manto 2022). Age‐related changes have been shown in the cerebellum and cerebral cortices only at the macroscopic level by in vivo studies, whereas microstructural changes have been mostly observed ex vivo throughout life (Andersen, Gundersen, and Pakkenberg 2003), and in patients with diseases progressing with aging (Grimaldi and Manto 2013; Louis et al. 2014). These studies showed different results, with loss of white matter (WM) up to 25% associated with loss of Purkinje and Granule cells (Andersen, Gundersen, and Pakkenberg 2003; Arleo et al. 2024) and thinning of dendritic trees of Purkinje cells (Louis et al. 2014).

Magnetic resonance imaging (MRI) studies have shown global macrostructural changes (volume loss) of gray matter (GM) and WM in the brain with aging (Andersen, Gundersen, and Pakkenberg 2003; MacDonald and Pike 2021; Walhovd et al. 2005); cortical thinning in the cerebral cortex (Sowell, Thompson, and Toga 2004) with prefrontal and frontal cortices (alongside hippocampus) most affected during aging (Jernigan et al. 2001); and loss of GM in the cerebellar cortex (Stalter et al. 2023).

Diffusion‐weighted MR imaging (dMRI) is a powerful and widely used imaging tool to quantify human brain microstructure in vivo and non‐invasively (Alexander et al. 2019; Jones 2010). Recent dMRI studies investigating variations of diffusion metrics with age observed a significant increase of mean diffusivity and decrease of fractional anisotropy in the cerebral cortex and subcortical regions (Helenius et al. 2002; Pfefferbaum et al. 2010; Raghavan et al. 2021; Schilling et al. 2022; Watanabe et al. 2013), while others remained inconclusive regarding the cerebellum (Behler, Kassubek, and Müller 2021; van Aalst et al. 2022).

Although very sensitive to microstructural changes, dMRI cannot unambiguously inform on changes in cellular composition due to the poor cell‐type specificity of water molecules. In contrast, diffusion‐weighted MRS (dMRS) provides higher cell‐type specificity (Cao and Wu 2017; Ligneul et al. 2024; Palombo et al. 2016, 2018; Palombo, Ligneul, and Valette 2017; Ronen and Valette 2015; Vincent, Palombo, and Valette 2020), offering the opportunity to inform on alterations of both cellular composition and microstructure with age, through the interpretation of measurements of metabolite diffusion properties. Some of the major brain metabolites are purely intracellular (e.g., N‐Acetyl‐aspartate, NAA; creatine and phosphocreatine, tCr, and choline compounds, tCho) and cell‐type specific (e.g., NAA mostly concentrated in neurons and tCho mostly concentrated in glia) and can be used to infer compartment specific microstructural changes (Ligneul et al. 2019, 2024; Palombo et al. 2016, 2018; Palombo, Ligneul, and Valette 2017). Previous dMRS studies focusing on aging reported on the changes in the apparent diffusion coefficient (ADC) of major brain metabolites across various brain regions in both healthy and pathological conditions (Branzoli et al. 2016; Deelchand et al. 2020; Zheng et al. 2012). Deelchand et al. investigated the five major intracellular metabolites' (tCr, tCho, Glutamate, myo‐Inositol and NAA) ADC and T_2_ dependence on healthy aging (*N* = 32 young adults versus *N* = 26 older adults) in the occipital, posterior and prefrontal cortices and concluded that the metabolite ADCs at short echo time (TE = 21.22 ms) was faster in healthy older adults and depended on the brain region, suggesting region‐specific alterations in the intra‐cellular microenvironment (Deelchand et al. 2020). However, it is still unknown how other informative diffusion properties of brain metabolites diffusion beyond the ADC change with aging. For example, the apparent diffusional kurtosis, a higher‐order diffusion metrics that quantifies the degree of non‐Gaussianity, could inform on the effect of restrictions and hinderance imposed by the microenvironment on the diffusion of intracellular metabolites (Jensen et al. 2005).

This work aims to fill this gap and provide first age‐trajectories of higher‐order diffusion properties of major intracellular metabolites (total N‐acetyl‐aspartate, tNAA: NAA + N‐acetyl‐aspartyl‐glutamate, NAAG; tCho: glycero‐phosphoryl‐choline, GPC + phosphoryl‐choline, PCho; and tCr: Cr, + PCr) and to highlight potential microstructural changes with age in the cerebral and cerebellar GM using dMRS. We focused our investigation on the cerebral and cerebellar cortices due to several factors: the cerebellum's role in mediating cognitive functions affected by brain aging, the greater complexity of dMRS/dMRI signals in the cerebellum compared to the cerebral cortex, and the cerebellum's unique cellular microstructure, including highly arborized, spiny Purkinje cells and Bergmann glia.

## 2 Material and Methods

### 2.1 Subjects

**FIGURE 1:**
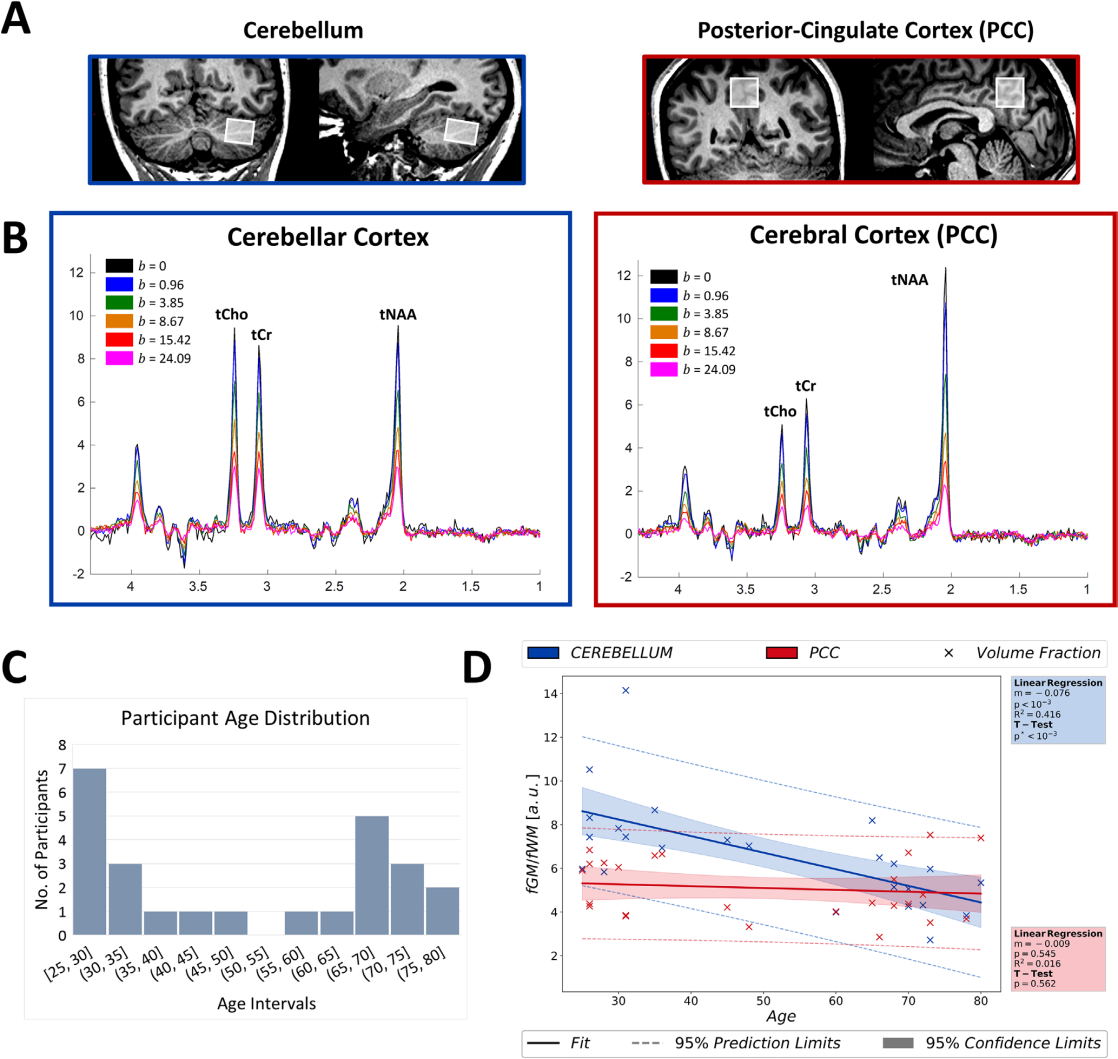
(A) Regions of interest are demonstrated on T_1_‐weighted images. (B) Diffusion‐weighted spectra are illustrated for both regions of interest; cerebellum (blue frame, left) and PCC (red frame, right). Direction averaged dMRS signals exhibit excellent spectral quality. Color‐coding in the legends displays b‐values in the units of ms/μm^2^. (C) Histogram of participants' age with an interval of five years. (D) Age‐trajectories of $\frac{\mathrm{fGM}}{\mathrm{fWM}}$ ratio in both ROIs and the results of statistical analyses reporting only a significant decrease in $\frac{\mathrm{fGM}}{\mathrm{fWM}}$ in the cerebellum with age. (*p < 0.00833 indicates statistical significance for the *t*‐test). fGM, gray matter volume fraction; fWM, white matter volume fraction; PCC, posterior cingulate cortex; ROI, region of interest; tCho, total choline; tCr, total creatine; tNAA, total N‐Acetyl‐aspartate.

A cohort of 25 healthy adults consisting of 11 females and 14 males were recruited for this study. The age range of the participants spanned from 25 to 80 years, with a mean age of 50.2 years and a standard deviation of 20.2 years. Dividing the cohort into younger (< 50 years) and older (> 50 years) adults, we have 13 participants (6 females) with a mean age of 31.8 and a standard deviation of 7.1 years, and 12 participants (5 females) with a mean age of 70.2 and a standard deviation of 5.3 years, respectively. The age distribution of the subjects is shown in the Figure 1C. The inclusion criteria for healthy participants were: (i) no known history of neurological or psychiatric conditions, (ii) no current treatments at the time of the study, with any prior treatments completed at least 2 weeks before recruitment, (iii) age greater than 18, and (iv) no contraindications related to MRI safety. No cognitive tests were conducted to confirm that the participants were cognitively intact. All subjects provided informed consent according to local procedures prior to the study. The study was approved by the local ethics committee.

### 2.2 Data Acquisition and Processing

dMRS data were acquired using a 3T Siemens Prisma scanner (Siemens Healthineers, Erlangen, Germany) with a 64‐channel receive‐only head coil at the Paris Brain Institute (Institut du Cerveau, ICM), France. Three‐dimensional T_1_‐weighted magnetization‐prepared rapid gradient echo images (field of view, 256 (anterior—posterior) × 256 (foot—head) × 231 (right—left) mm^3^); isotropic resolution, 0.9 mm; repetition and echo time (TR/TE), 2300/2.08 ms; total acquisition time, 5 min. 17 s. were acquired to position the spectroscopic region‐of‐interest (ROI) and to perform tissue segmentation. Two ROIs targeting GM in the cerebellum and posterior‐cingulate‐cortex (PCC) were examined using a diffusion‐weighted semi‐LASER sequence (Genovese et al. 2021). The ROIs were defined as 5.3 cm^3^ (15 × 16 × 22 mm^3^) in the cerebellum and 8.0 cm^3^ (20 × 20 × 20 mm^3^) in the PCC to maximize GM volume fraction (above 70%) in both ROIs. Spectral data was recorded with a spectral bandwidth of 3000 Hz and complex data points of 2048 at TE of 125 ms. During measurements, pulse triggering was applied and maintained the average TR at three cardiac cycles. Hence, the average acquisition time per ROI was around 25 min on average. Diffusion‐weighting was applied using tetrahedral‐encoding scheme in directions of (−1 −1 −1), (−1 1 1), (1 −1 1), and (1 1 −1). Six *b*‐values (b = [0.01, 1.012, 4.03, 9.06, 16.09, 25.1] ms/μm^2^) were applied with an effective gradient duration (δ) of 26.4 ms (two pairs of bipolar gradients with 6.6 ms duration) and an effective diffusion gradient separation (Δ) of 62.5 ms. The effective b‐values were computed by including crusher and slice selection gradients as well as cross‐terms compensation. The estimated coefficient of variation of b value across direction was consistently < 3%, justifying averaging across direction with minimal negligible bias. Twenty‐four transients were acquired for each diffusion‐weighted condition and saved individually for further postprocessing. Water suppression was performed using variable power with optimized relaxation delays (VAPOR) and outer volume suppression (Tkac et al. 1999). The water suppression flip‐angle was calibrated for each participant. Additionally, water signals were acquired using the same diffusion‐weighted conditions for eddy‐current correction, excluding ultra‐high b‐values due to poor water signal. B_0_ shimming was performed using a fast automatic shimming technique with echo‐planar signal trains utilizing mapping along projections, FASTESTMAP (Gruetter and Tkac 2000).

Spectral processing was performed by following the state‐of‐the‐art guidelines (Ligneul et al. 2024) on MathWorks MATLAB R2022a (The MathWorks Inc. 2022).

Zero‐order phase fluctuations and frequency drifts were corrected on single transients before averaging using the NAA peak. A peak‐thresholding procedure was applied, for each diffusion condition, to discard the transients with artefactual low signal‐to‐noise ratio (SNR) caused by non‐translational tissue motion (Genovese et al. 2021). After processing of the transients in an acquisition, for example, a *b*‐value measurement in a diffusion direction, the transients were averaged for independent data fitting.

GM, WM, and cerebrospinal fluid (CSF) volume fractions were calculated in the ROIs using the T_1_‐weighted images and the segment tool of SPM12 and MATLAB routines.

### 2.3 Data Fitting

For each diffusion‐weighted condition, averaged spectra were fitted independently with LCModel (Provencher 1993). The SNR of spectra was reported from LCModel's output (i.e., the ratio between signal intensity at 2.01 ppm and twice the root mean square of fit residuals) together with Cramer‐Rao lower bounds (CRLB).

The basis set was simulated with an in‐house written routine in MATLAB based on the density matrix formalism (Henry et al. 2006) and using previously reported chemical shifts and *J*‐couplings (Govindaraju, Young, and Maudsley 2000; Kaiser et al. 2010). The basis set included ascorbate, aspartate, Cr, γ‐aminobutyric acid, glucose, glutamate, glutamine, glutathione, GPC, *myo*‐inositol, lactate, NAA, NAAG, PCr, PCho, phosphorylethanolamine, scyllo‐inositol, and taurine. Independent spectra for the CH_3_ and CH_2_ groups of NAA, Cr, and PCr were simulated and included in the basis set.

### 2.4 Data Analysis

To characterize the metabolites' higher order diffusion properties, multiple diffusion signal analyses were conducted including diffusion signal representations and biophysical models (Jensen et al. 2005; Ligneul et al. 2024; Palombo, Ligneul, and Valette 2017). All the data and analysis codes underpinning the results presented here can be found upon publication in the Cardiff University data catalog and on Github: https://github.com/kdrsimsek/aging_dMRS_project.

#### 2.4.1 dMRS Signal Representations

First, the direction‐averaged diffusion signals were fitted monoexponentially up to *b* < 5 ms/μm^2^ to estimate the apparent diffusion coefficient (ADC) and characterize Gaussian properties (Ligneul et al. 2024). Kurtosis signal representation (from Equation 5 in (Jensen et al. 2005)) was used to estimate the apparent diffusion kurtosis (K) and determine non‐Gaussian properties of metabolites up to b < 10 ms/μm^2^ (Genovese et al. 2021).

#### 2.4.2 dMRS Biophysical Models

For biophysical modeling, the astro‐sticks model was fitted to the direction‐averaged signals at all *b*‐values to estimate the apparent intra‐stick axial diffusivity ($D_{\text{intra}}$) (Ligneul et al. 2024; Panagiotaki et al. 2012)

(1) $$\frac{S}{S_{0}}=\int_{0}^{1}e^{-bD_{\text{intra}}\cos^{2}\theta }d\left(\cos\theta \right)=\frac{\sqrt{\pi }}{2}\frac{\mathrm{erf}\left(\sqrt{bD_{\text{intra}}}\right)}{\sqrt{bD_{\text{intra}}}}$$

here, the equation describes direction‐averaged diffusion signal for the astro‐sticks model. θ is the angle between the main axis of a given stick and the applied diffusion gradient. Additionally, astro‐sticks model was modified to incorporate an effective intra‐stick axial diffusivity ($D_{\mathrm{eff}}$) defined as (Palombo, Ligneul, and Valette 2017; Palombo et al. 2018; Sukstanskii and Yablonskiy 2008; Yablonskiy and Sukstanskii 2010):

(2) $$D_{\mathrm{eff}}\left(D_{\text{intra}},K_{\text{intra}},b,\theta \right)=D_{\text{intra}}\left(1-K_{\text{intra}}D_{\text{intra}}b\cos^{2}\theta \right)$$

here, $K_{\text{intra}}$ is the apparent intra‐neurite axial kurtosis and quantifies non‐Gaussian diffusion characteristics stemming from hindering or restricting structures randomly displaced along the cellular processes, such as dendritic spines (Palombo et al. 2018; Sukstanskii and Yablonskiy 2008; Yablonskiy and Sukstanskii 2010). The corresponding powder‐averaged signal for the modified astro‐sticks model is computed by numerical integration given in the following equation:

(3) $$S/S_{0}=\int_{0}^{1}e^{-bD_{\mathrm{eff}}\cos^{2}\theta }d\left(\cos\theta \right)$$

#### 2.4.3 Fitting Routine

Data analysis was conducted within the Python programming environment. Following spectral quantification using LCModel, we estimated the diffusion‐weighted signal amplitude from the area under each metabolites' peak(s) and direction‐averaged it at each b value to obtain the direction‐averaged diffusion signal decay for each metabolite. Diffusion fitting was performed using Levenberg–Marquardt non‐linear least squares optimization in the Python library ‘lmfit’ (https://pypi.org/project/lmfit/). No constraints were imposed on the modeling functions, but boundary conditions of each model parameter were defined to be positive and not to exceed free metabolites' diffusivity 1.0 μm^2^/ms (Döring et al. 2018) and 3.0 for apparent kurtosis parameters (Jensen et al. 2005). Three major metabolites were examined: tNAA as a neuronal biomarker; tCho as a glial biomarker; and tCr as a biomarker comprised in both neuronal and glial cells. Notably, one dataset in the cerebellum, acquired from an older subject, suffered from very poor SNR; hence, excluded from the whole analysis from the start.

### 2.5 Statistical Analysis

Linear regression was performed on all estimated parameters to determine age‐trajectories with computed 95% confidence interval and prediction limits. To analyze the specific impact of age on the changes of diffusion metrics, a regression analysis with age as the independent variable and each estimated model parameter as the dependent variable was performed, also accounting for $\frac{\mathrm{fGM}}{\mathrm{fWM}}$ (the ratio between GM and WM volume fractions) as covariate by fitting the following expression: $y∼\beta_{0}+\beta_{1}\cdot \mathrm{age}+\beta_{2}\cdot \frac{\mathrm{fGM}}{\mathrm{fWM}}$. Additionally, an independent *t*‐test between younger (age < 50) and older (age ≥ 50) people was performed to assess statistically significant differences between younger and older adult groups. Bonferroni correction was applied for only *t*‐test, including two brain regions and three metabolites for each diffusion metric and the *p*‐value, the threshold for statistical significance, was redefined to be 0.0083 (0.05/6). In both statistical analyses, the model parameters' values converging to the lower bound in the fitting were excluded from the age‐trajectory analysis because considered unreliable.

## 3 Results

To simplify inspection of the findings, a color‐coding scheme is used to identify the cerebellum and the PCC results as blue and red more clearly, respectively.

Exemplary diffusion‐weighted spectra acquired from both brain regions are shown in Figure 1B which exhibit good spectral quality—linewidths at b_0_/b_max_: 4.17/4.84 Hz in the cerebellum and 3.30/4.67 Hz in the PCC. SNRs obtained from the corresponding LCModel fit results were 18 ± 3 and 24 ± 4 (mean ± standard deviation over all subjects) at b=0 (i.e., no diffusion‐weighting) and 7 ± 2 and 6 ± 2 at the highest b value in the cerebellar and cerebral cortexes, respectively. The tissue volume fractions (mean ± standard deviation over all subjects) were as follows: fGM: 0.82 ± 0.05 (GM volume fraction), fWM: 0.12 ± 0.05 (WM volume fraction), and fCSF: 0.06 ± 0.03 (CSF volume fraction) in the cerebellum; fGM: 0.69 ± 0.07, fWM: 0.14 ± 0.03, and fCSF: 0.17 ± 0.08 in the PCC. The localizations of spectroscopic voxels in both ROIs are depicted in Figure 1A. Furthermore, the age‐trajectory for $\frac{\mathrm{fGM}}{\mathrm{fWM}}$ ratio was investigated for variations with age and reported in Figure 1D. A significant decrease (~17%) of fGM with age in the cerebellum, while a small (~4%) not significant change in the PCC was observed.

The CRLB obtained from LCModel fit was used to assess the quality of the quantification but not as an exclusion criterion. Overall fit results are excellent with low CRLBs (< 5%) in both ROIs for the non‐diffusion weighted spectra. For the diffusion‐weighted spectra, we quantified metabolites' areas for each b value and each direction, resulting in a CRLB value per direction. Here, as a summary measure, we report the mean of the estimated CRLBs across diffusion directions at the highest b values: CRLB_tNAA_ = 4%, CRLB_tCho_ = 6%, and CRLB_tCr_ = 4% in the cerebellum and CRLB_tNAA_ = 7%, CRLB_tCho_ = 10%, and CRLB_tCr_ = 5% in the PCC.

### 3.1 Metabolite Diffusion Properties

**FIGURE 2:**
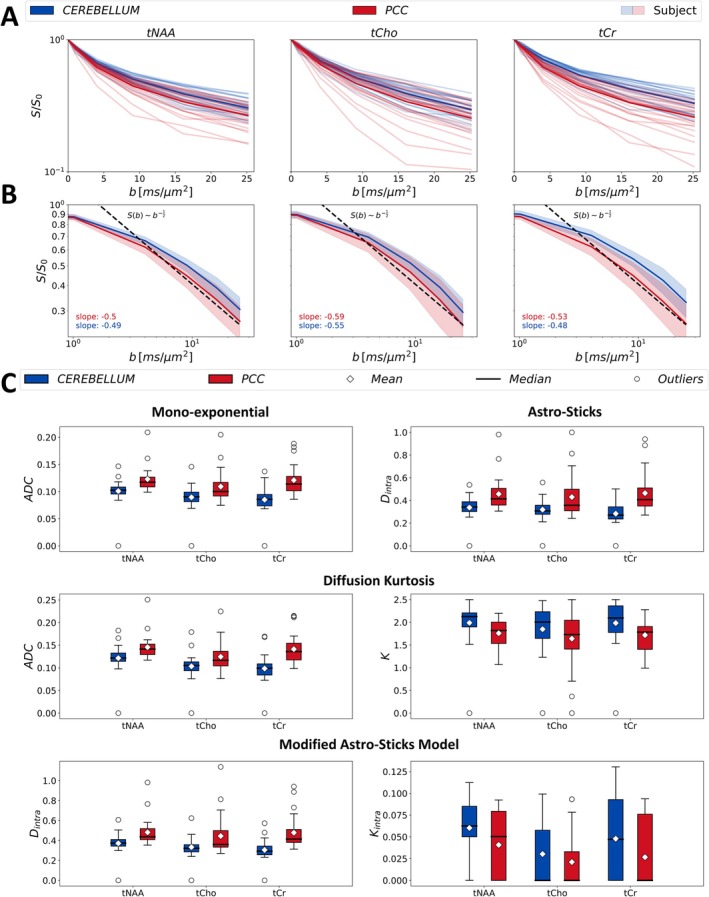
(A) Diffusion signals of tNAA, tCho, and tCr obtained from each subject (light) and cohort averaged signals (dark) are illustrated in the figure for both brain regions: Cerebellum (blue) and posterior‐cingulate‐cortex (red). (B) Comparison between the cohort averaged signal decays at the highest b‐values with the characteristic $b^{-1/2}$ scaling (dashed black lines) of the astro‐sticks models. Error bands denote the standard deviation across subjects. We report the fitted slope of the log(S) versus log(b) for each metabolite and ROI. (C) The estimated parameters of each metabolite by mono‐exponential, kurtosis representations and astro‐sticks and modified astro‐sticks models from each subject are illustrated in the box‐and‐whiskers plot for both region of interests: Cerebellum (blue) and PCC (red). ADC: Apparent diffusion coefficient; *D*_intra_, apparent intra‐neurite axial diffusivity; K, apparent diffusion kurtosis; *K*_intra_, apparent intra‐neurite axial kurtosis; PCC, posterior cingulate cortex; tCho, total choline; tCr, total creatine; tNAA, total N‐Acetyl‐aspartate.

Metabolite diffusion signals obtained from all subjects are displayed in Figure 2A for both cerebellum and PCC. The diffusion signals obtained from all participants (light) are reported alongside the corresponding cohort averages (dark). Figure 2B compares the cohort averaged diffusion signal decays at the highest b‐values with the characteristic scaling ($∼b^{\frac{1}{2}}$) of the astro‐sticks models and displays the corresponding slopes for all metabolites in both ROIs. A mildly faster decay was observed in the glial biomarker tCho. Overall, slower metabolite diffusion was observed for all metabolites in the cerebellum compared to PCC. Figure 2C presents the results of the estimated diffusion parameters from all subjects as a box‐whiskers plot for all signal representations and biophysical models. The corresponding mean values of the estimated parameters obtained from the cohort are charted in Table 1. In the cerebellum, the model parameters for one dataset could not be estimated (and highlighted as an outlier with values of zero), due to low SNR at higher b‐values. In all cases, the estimated apparent diffusivities (ADCs & $D_{intra}$) are lower in the cerebellum than in the PCC. Correspondingly, the kurtosis estimates (K & $K_{\text{intra}}$) are higher in the cerebellum than in the PCC, for all metabolites. Noticeably, $K_{\text{intra}}$ of tCho and tCr in both ROIs exhibit high variability due to relatively higher CRLB; e.g. in the tCho results, the median values in each metabolite result are at the lower bound while the mean values are higher as shown in Figure 2C.

**TABLE 1:** Estimated model parameters obtained from cohort averaged diffusion signals are charted with the corresponding error values in the fit (estimation ± error).

ROI	Fit	Parameter	tNAA	tCho	tCr
CEREBELLUM	Monoexp	ADC	0.105 ± 0.007	0.093 ± 0.004	0.088 ± 0.004
	Kurtosis	ADC	0.126 ± 0.006	0.107 ± 0.002	0.103 ± 0.002
		K	2.108 ± 0.107	1.992 ± 0.077	2.197 ± 0.072
	Astro‐sticks	$D_{intra}$	0.346 ± 0.012	0.325 ± 0.007	0.287 ± 0.007
	Modified Astro‐sticks	$D_{intra}$	0.384 ± 0.015	0.325 ± 0.022	0.311 ± 0.011
		$K_{intra}$	0.071 ± 0.014	—	0.063 ± 0.018
PCC	Monoexp	ADC	0.122 ± 0.005	0.108 ± 0.002	0.120 ± 0.003
	Kurtosis	ADC	0.145 ± 0.002	0.124 ± 0.001	0.141 ± 0.002
		K	1.767 ± 0.034	1.718 ± 0.027	1.732 ± 0.028
	Astro‐sticks	$D_{intra}$	0.440 ± 0.006	0.407 ± 0.012	0.440 ± 0.006
	Modified Astro‐sticks	$D_{intra}$	0.468 ± 0.009	0.407 ± 0.043	0.440 ± 0.021
		$K_{intra}$	0.046 ± 0.011	—	—

*Note:* All signal representations and biophysical model results are tabulated in the table. “—” indicates that the estimations converge to zero.

Abbreviations: ADC: Apparent diffusion coefficient; *D*_intra_, apparent intra‐neurite axial diffusivity; *K*, apparent diffusion kurtosis; *K*_intra_, apparent intra‐neurite axial kurtosis; PCC, posterior cingulate cortex; tCho, total choline; tCr, total creatine; tNAA, total N‐Acetyl‐aspartate.

### 3.2 Age‐Trajectories

**FIGURE 3:**
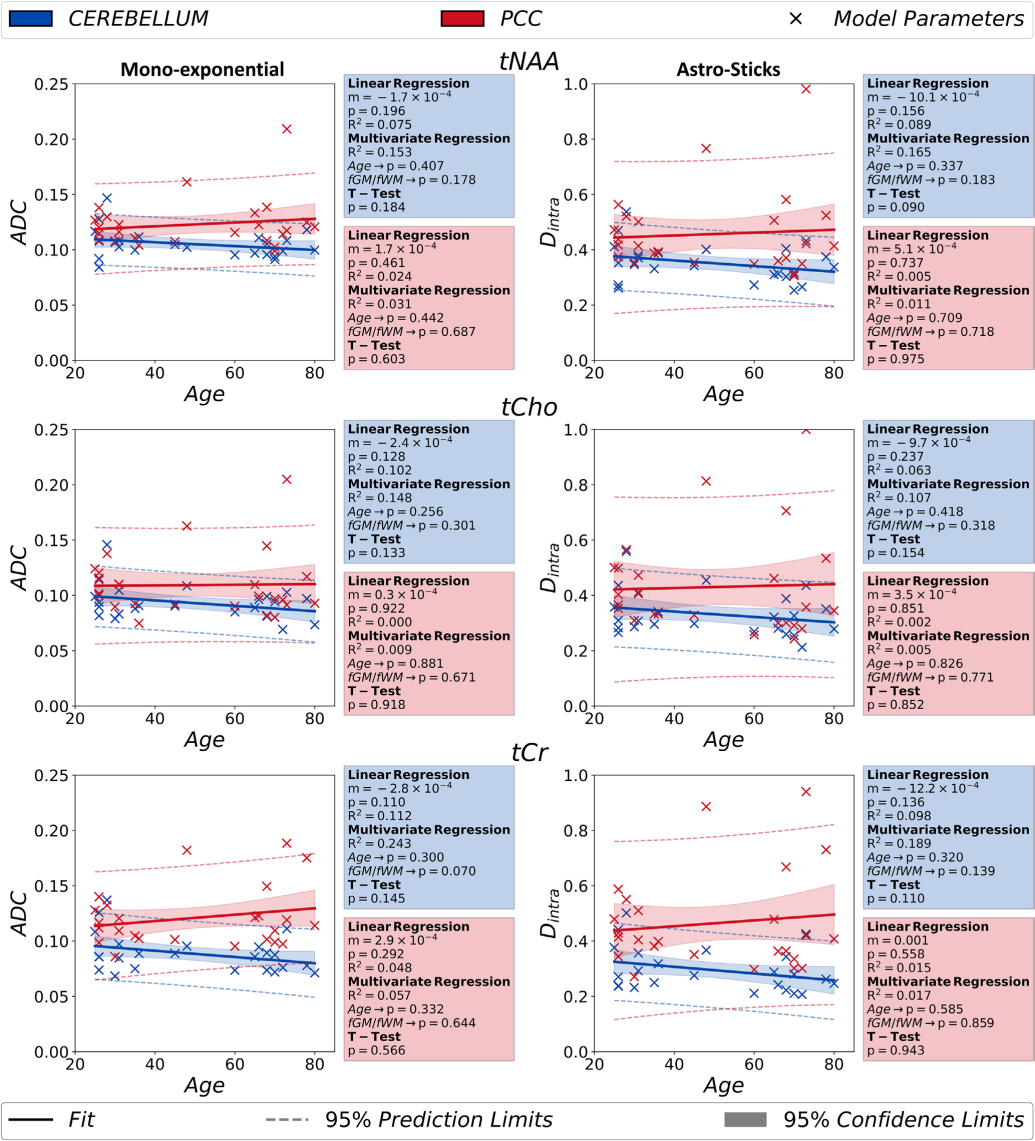
The results obtained from monoexponential signal analysis (*b* < 5 ms/μm^2^) (ADC) and astro‐stick model ($D_{\text{intra}}$) are documented in the figure. The independent *t*‐test analyses performed between younger (age < 50) and older groups (age ≥ 50) do not report any statistically significant change in these parameters with aging. The p‐value in linear regression is a measure for how significant the estimated slope is in the analysis. (*p < 0.00833 indicates statistical significance for the *t*‐test). ADC: Apparent diffusion coefficient; *D*_intra_, apparent intra‐neurite axial diffusivity; fGM, gray matter volume fraction; fWM, white matter volume fraction; PCC, posterior cingulate cortex; tCho, total choline; tCr, total creatine; tNAA, total N‐Acetyl‐aspartate.

The age‐trajectories for apparent diffusivities (ADC & $D_{\text{intra}}$) of monoexponential representation and astro‐sticks model are grouped together and presented in Figure 3. The apparent diffusivities presented similar trends with age for all metabolites: increasing in the PCC and decreasing in the cerebellum.

**FIGURE 4:**
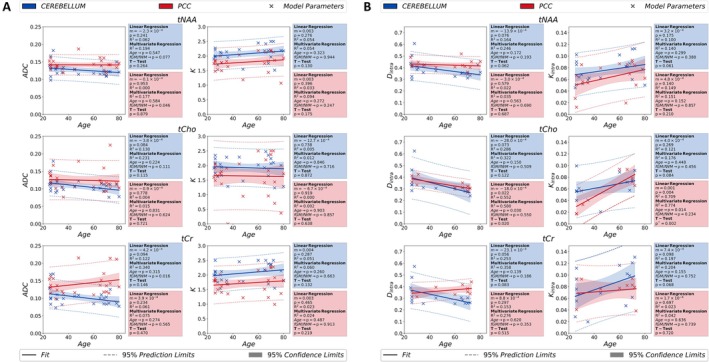
Age dependences of the estimated model parameters for kurtosis (ADC & K) in (A) and modified astro‐stick model ($D_{\text{intra}}$ & $K_{\text{intra}}$) in (B), obtained from studied metabolite signals, are depicted in the figure. For each brain region, a linear regression, a regression analysis using age and $\frac{\mathrm{fGM}}{\mathrm{fWM}}$ as independent and dependent variables, respectively, and a paired *t*‐test between two groups (age < 50 and age ≥ 50) are performed to analyze the impact of age and tissue composition on the estimated parameters. For statistical tests, the confidence and prediction limits are also depicted in the figure. (*p < 0.00833 indicates statistical significance for the *t*‐test). ADC: Apparent diffusion coefficient; *D*_intra_, apparent intra‐neurite axial diffusivity; fGM, gray matter volume fraction; fWM, white matter volume fraction; K, apparent diffusion kurtosis; *K*_intra_, apparent intra‐neurite axial kurtosis; PCC, posterior cingulate cortex; tCho, total choline; tCr, total creatine; tNAA, total N‐Acetyl‐aspartate.

The age‐trajectories for diffusion kurtosis (ADC & K) and modified astro‐sticks model ($D_{\text{intra}}$ & $K_{\text{intra}}$) analyses were grouped together and showed in Figure 4. The age‐trajectories for the diffusion kurtosis parameters depicted in Figure 4A predominantly show similar trends for all metabolites except for the ADC of tCr in the PCC and K of tCho in the cerebellum, which exhibit an opposite trend. Likewise, the age‐trajectories of modified astro‐sticks model parameters show a decreasing trend in $D_{\text{intra}}$ and an increasing trend in $K_{\text{intra}}$ for all metabolites in both ROIs as illustrated in Figure 4B. The only exception is the $D_{\text{intra}}$ of tCr in the PCC showing an increasing trend.

Overall, the statistical analyses performed over diffusion metrics of tNAA (the neuronal biomarker), tCho (glial biomarker) and tCr (less cell‐type specific) do not report any significant change with age for all the higher‐order diffusion metrics investigated in this study (*p* > 0.05). Notably, the *t*‐test results of tCho $K_{\text{intra}}$ show only a significant increase in the PCC. Considering the high noise level in tCho and the median value of tCho $K_{\text{intra}}$ at the lower bound in the modified astro‐sticks model fitting, this outcome needs to be treated carefully.

## 4 Discussion

This work investigates variations in the higher‐order diffusion properties of major intracellular brain metabolites with healthy aging in the cerebral and cerebellar GM in vivo in the human brain using dMRS and clinical 3T MRI scanner.

### 4.1 Metabolites Apparent Diffusivity in Cerebellar and Cerebral GM

Apparent diffusivities (ADC & $D_{\text{intra}}$) of the studied metabolites agree with literature findings (Branzoli et al. 2014; Deelchand, Auerbach, and Marjańska 2018; Döring et al. 2018; Döring and Kreis 2019; Ingo et al. 2018; Kan et al. 2012; Najac et al. 2016; Palombo et al. 2016; Şimşek et al. 2022). Relatively slower metabolite apparent diffusivities in the cerebellum might stem from higher microstructural complexity of cellular composition compared to PCC: the Purkinje and granule cells are highly abundant in the cerebellum (Louis et al. 2014) while the PCC is comprised by the less complex Pyramidal neurons. The higher values in a few metabolites' ADC estimates in PCC (outliers in Figures 2C, 3 and 4A) is most likely due to the lower SNR of the corresponding datasets.

### 4.2 Age‐Dependence of Metabolites Apparent Diffusivity

Overall, estimated apparent diffusivities did not present any significant trend nor changes with age, in contrast to mono‐exponential ADCs reported by the only study in the literature (Deelchand et al. 2020). This difference might originate from having a different sample size and more likely from different ROI tissue volume composition. In contrast to our work, Deelchand et al. recruited more participants, in two age groups (*N* = 32 young: 18–22 and *N* = 26 old: 70–83 years old); investigated two more metabolites (Glu and mI); and reported on T_2_ relaxation dependence on aging. Regarding distinctions in tissue composition, the WM content in the PCC ROI in our work is around half that in the Deelchand's work (our work, fWM = 14%; Deelchand's work, fWM ≃ 30%). Higher fGM in our ROIs leads to a more isotropic microenvironment for metabolite diffusion; thus, a weaker dependence of metabolite apparent diffusivity on the fiber orientation. Other contributing factors might be differences in diffusion times (~50 ms in our work and 118 ms in Deelchand's work) and encoding schemes. Previous studies have shown that diffusion times have strong effects on estimated ADCs in both GM and WM (Assaf and Cohen 1998; Döring and Kreis 2019; Ligneul, Palombo, and Valette 2017; Ligneul and Valette 2017), while TE‐dependence of metabolites ADC was only significant in ROIs with high content of WM (Branzoli et al. 2014) like in Deelchand's work (TE = 21.2 ms) and was negligible for ROIs with high content of GM (Ligneul, Palombo, and Valette 2017), like in our study.

### 4.3 Metabolites Apparent Kurtosis and Non‐Gaussianity in Cerebellar and Cerebral GM

Estimated metabolite diffusion kurtosis K values agree with current literature (Döring et al. 2023; Genovese et al. 2021; Ingo et al. 2018; Mougel, Valette, and Palombo 2023). In accordance with metabolite apparent diffusivities, the K & $K_{intra}$ for all metabolites in the cerebellum compared to PCC agree with the expected higher complexity of the cellular microenvironment. For instance, the Purkinje cells in the cerebellum have higher spine density and higher branching order (Santamaria et al. 2006) in contrast to the PCC, which comprises mostly Pyramidal cells with lower spine density and branching order (Holtmaat et al. 2005). Therefore, the higher microstructural complexity in the cerebellum might lead to higher tNAA (the neuronal biomarker) apparent K & $K_{intra}$. Additionally, the relatively higher K & $K_{intra}$values for tCho (glial biomarker) in the cerebellum might be due to the presence of highly arborized Bergmann glia (Sild and Ruthazer 2011). The same rationale can explain the observed lower diffusivities in the cerebellum compared to the PCC.

The functional form of the signal at the highest b‐values supports the choice of the astro‐sticks models (see Figure 2B), since the signal consistently scales as $∼b^{-1/2}$. Notably, for tCho, we observed a slight deviation toward a faster decay ($∼b^{-0.6}$), potentially indicating a modest influence of non‐negligible fiber calibers associated with glial processes (Palombo, Ligneul, and Valette 2017).

### 4.4 Age‐Dependence of Metabolites Apparent Kurtosis and Non‐Gaussianity

The age‐trajectories of metabolite diffusion properties reveal overall similar trends for apparent diffusivities (ADC & $D_{\text{intra}}$) from signal representations and biophysical models. The significant increase with age in $K_{\text{intra}}$ of tCho in the PCC requires cautious interpretation due to the low SNR leading to incompatibility in the model fitting (i.e., higher‐order term converges to the lower‐bound). Figure 2C illustrates that the median value of tCho $K_{intra}$ is at the lower bound. Therefore, the low SNR in tCho might cause instability in fitting of modified astro‐sticks model that resulted in a significant increase in $K_{\text{intra}}$ in the PCC.

The observed overall decrease in diffusivities and increase in the non‐Gaussianity of tNAA (and tCr) align with histological evidence (McElroy et al. 2024) showing that, with healthy aging, Purkinje cell somas can shrink by up to 33% in volume (Andersen, Gundersen, and Pakkenberg 2003), alongside restructuring of their dendritic trees (Hadj‐Sahraoui et al. 2001; Quackenbush, Ngo, and Pentney 1990; Zhang et al. 2006). Additionally, in cerebellar basket cells, there is an increase in Golgi volume, dense bodies, and ground substance, accompanied by a significant reduction in rough endoplasmic reticulum surface area (Henrique et al. 2001; Sturrock 1990). Together, these factors likely elevate the viscosity of the intracellular space and increase the restriction of metabolite diffusion, resulting in lower diffusivities and greater non‐Gaussianity.

### 4.5 Analysis of Potential Confounders: The Negligible Impact of ROI Tissue Composition

Our findings of ~4% decrease in $\frac{\mathrm{fGM}}{\mathrm{fWM}}$ in the PCC and ~ 17% decrease in $\frac{\mathrm{fGM}}{\mathrm{fWM}}$ in the cerebellum agree with evidence from the literature. For example, Mann et al. (Mann et al. 2011) found ~6% decrease in the whole cingulate GM volume between decades 20–80s. Bernard and Seidler (Bernard and Seidler 2013) found that young adults have larger cerebellar GM volume than older adults, with Crus I (where the majority of our spectroscopic voxel is located) having up to ~15% decrease in GM volume. The reason why cerebellar GM declines more with age than cerebral GM remains a topic of debate in the literature; however, investigating this issue is beyond the scope of our study.

The multivariate regression analysis does not report any significant impact of the accounted variables age (as independent) and $\frac{\mathrm{fGM}}{\mathrm{fWM}}$ (as dependent) on the variation of diffusion metrics (*p* > 0.05). Hence, the trend in age‐trajectory cannot be attributed to changes in the volume fractions of tissue compositions in the ROIs despite the slight underestimations of fWM in the cerebellum (< 5%) over all participants. The only exception is for the tCho $K_{\text{intra}}$ in the PCC (Figure 4B), having *p*‐value for age just below the threshold (*p* = 0.013). However, the observed change possibly arises from the encountered model fitting issues in tCho, the glial biomarker.

A previous study reported that the ADC of tNAA changes by 8% between young and old groups and argued that the contribution stemming from their ROIs tissue composition would be relatively small in comparison to the observed percentage change in the tNAA ADC (Deelchand et al. 2020). A similar argument can be made in our study. For instance, $D_{\text{intra}}$ of tCr from the astro‐sticks model exhibits the strongest change of about 10% increase within the age limit in the PCC (Figure 4B). However, the change in the ROI tissue composition, $\frac{\mathrm{fGM}}{\mathrm{fWM}}$, is only around 2% (Figure 1C) and cannot alone explain the changes observed in the PCC. Moreover, the multivariate analysis of the corresponding age‐trajectory does not demonstrate any dependence on $\frac{\mathrm{fGM}}{\mathrm{fWM}}$ (*p* > 0.05). Therefore, other factors, more directly linked to changes in the tissue microstructure and cellular composition might explain the observed trends in age‐trajectories. A longitudinal study monitoring microstructural alterations in stroke linked an increase of tCr ADC with astrogliosis and glial reactivity in the presence of neuroinflammation in stroke patients (Genovese et al. 2023). Accordingly, an increase in astrogliosis and glial reactivity with aging was also reported in the literature (Cotrina and Nedergaard 2002) that might explain the slight increasing trend in the $D_{\text{intra}}$ of tCr.

### 4.6 Limitations

Our study has a few limitations that future studies may want to address. Although exploring additional brain regions beyond the PCC and cerebellum would have been valuable, the extended acquisition time required by our advanced dMRS protocol limited our ability to examine further areas. Nonetheless, investigating alterations in the cellular microstructure of cerebellar GM is particularly promising for clinical applications, such as the early diagnosis and treatment planning of conditions like essential tremor (Louis et al. 2014). Diffusion‐weighted spectra are very sensitive to the bulk or physiological motion occurring during the acquisition, causing variations in signal amplitude and phase (Branzoli et al. 2014; Döring et al. 2018; Ligneul et al. 2024; Şimşek et al. 2022). Employing cardiac triggering during measurements and performing SNR thresholding partially eliminated these (Genovese et al. 2021; Ligneul et al. 2024). Due to poor water signal at high b‐values, eddy‐current correction was not applied to the spectra acquired at ultra‐high b values. However, we investigated the effect of ECC correction on the highest and lowest SNR datasets for the two lowest b‐values (~1 and ~4 ms/μm^2^) across all directions. The difference in tNAA signals with and without ECC remained below 5% for each direction and b‐value in both SNR cases. Another crucial limiting factor in this study is smaller sample size (25) – the sample size is 58 in the Deelchand's work, reducing the statistical power of the current study. Because of the relatively small sample size, the *t*‐test analysis was performed on two age groups (age < 50 and age ≥ 50) to accommodate enough datasets. We acknowledge that the tetrahedral encoding might be insufficient to encode diffusion isotropically at high *b*‐values in ROI with high anisotropy. However, the relative GM fraction in both of our ROIs is very high (89%) and the impact of a potential bias due to partial powder averaging is expected to be minor and negligible. Furthermore, across these four directions, no significantly faster or slower decay was observed, with only slight variations in diffusion signals between directions. Finally, our ability to include more directions at such ultra‐high b‐values was constrained by the maximum gradient strength of our clinical scanner (80 mT/m), which is why we had to utilize tetrahedral encoding to maximize diffusion weighting.

### 4.7 Importance and Potential Impact

The age‐trajectories here reported are a precious resource for the community because they provide reference values for a large set of diffusion properties in two brain regions of potential interest for many diseases (e.g., Alzheimer's disease and motor disorders), previously unavailable. As an example, choline compound is known as a neuroinflammation biomarker (De Marco et al. 2022; Genovese et al. 2021). A recent dMRS study (De Marco et al. 2022; Genovese et al. 2021) showed a significant increase in tCho ADC in the thalamus with neuroinflammation. The age‐trajectories reported here provide reference values for the healthy brain cerebellum and PCC, suggesting that the age‐related changes of tCho ADC are less than 10% (decrease in the cerebellum and increase in the PCC with age) which can help further interpreting tCho diffusivity results in studies of neuroinflammation in these brain regions. Age is often found to be a significant covariant in the analyses of the changes of metabolites' diffusivity. Here we show to what extent age indeed alter the diffusion properties of major metabolites in ROIs mostly comprised of GM (> 70%). For instance, for the widely used ADC index, no statistically significant changes are observed for tNAA, tCr and tCho between younger (< 50) and older (≥ 50) adults, with metabolites ADCs being overall less than 10% lower in older adults in the cerebellum, and less than 5% higher in older adults in the PCC.

It is important to note that our findings are primarily applicable to spectroscopic ROIs with a high GM content (> 80%). Studies have reported substantial differences in the diffusion properties of intracellular brain metabolites between gray and white matter in humans (Ercan et al. 2015; Lundell et al. 2021). Therefore, we do not expect our results to generalize to spectroscopic ROIs with markedly different tissue compositions. We suggest that future studies should explore age‐related changes in higher‐order diffusion metrics of metabolites in ROIs with a high WM content and/or a balanced mix of GM and WM.

## 5 Conclusion

This study offers previously unavailable age‐trajectories of major intracellular brain metabolites' diffusion properties in cerebral and cerebellar GM. We showed that observed variations in metabolite diffusion properties with healthy aging are minimal and most likely caused by age‐related microstructural changes, demonstrating the potential utility of the metabolites high‐order diffusion parameters as new (neuronal and glial) biomarkers of tissue pathology. The proposed age‐trajectories provide benchmarks for identifying anomalies in the diffusion properties of major brain metabolites, which could be related to pathological mechanisms altering both the GM microstructure and cellular composition.

## Author Contributions

Kadir Şimşek: conceptualization, formal analysis, methodology, investigation, writing – original draft. Cécile Gallea: writing – review and editing. Guglielmo Genovese: methodology, formal analysis; data curation, writing – review and editing. Stephane Lehéricy: writing – review and editing. Francesca Branzoli: formal analysis; funding acquisition, methodology, project administration, writing – review and editing. Marco Palombo: conceptualization, methodology, supervision, resources, project administration, investigation, writing – review and editing.

## Conflicts of Interest

The authors declare no conflicts of interest.

## Data Availability

All the data and analysis codes underpinning the results presented here can be found upon publication in the Cardiff University data catalog and on Github: https://github.com/kdrsimsek/aging_dMRS_project.
